# Cost-Effectiveness of Artificial Intelligence-Enabled Electrocardiograms for Early Detection of Low Ejection Fraction: A Secondary Analysis of the Electrocardiogram Artificial Intelligence-Guided Screening for Low Ejection Fraction Trial

**DOI:** 10.1016/j.mcpdig.2024.10.001

**Published:** 2024-10-26

**Authors:** Viengneesee Thao, Ye Zhu, Andrew S. Tseng, Jonathan W. Inselman, Bijan J. Borah, Rozalina G. McCoy, Zachi I. Attia, Francisco Lopez-Jimenez, Patricia A. Pellikka, David R. Rushlow, Paul A. Friedman, Peter A. Noseworthy, Xiaoxi Yao

**Affiliations:** a Robert D. and Patricia E. Kern Center for the Science of Health Care Delivery, Mayo Clinic, Rochester, MN; b Division of Preventive and Occupational Medicine, Mayo Clinic, Rochester, MN; c Department of Cardiovascular Medicine, Mayo Clinic, Rochester, MN; d Department of Family Medicine, Mayo Clinic, Rochester, MN; e School of Medicine, University of Maryland, Baltimore, MD

## Abstract

**Objective:**

To investigate the cost-effectiveness of using artificial intelligence (AI) to screen for low ejection fraction (EF) in routine clinical practice using a pragmatic randomized controlled trial (RCT).

**Patients and Methods:**

In a post hoc analysis of the electrocardiogram (ECG) AI-guided screening for low ejection fraction trial, we developed a decision analytic model for patients aged 18 years and older without previously diagnosed heart failure and underwent a clinically indicated ECG between August 5, 2019, and March 31, 2020. In the previously published RCT, the intervention arm underwent an AI-guided targeted screening program for low EF with a workflow embedded into routine clinical practice—AI was applied to the ECG to identify patients at high-risk and recommended clinicians to order an ECG and the control arm received usual care without the screening program. We used results from the RCT for rates of low EF diagnosis and a lifetime Markov model to project the long-term outcomes. Quality-adjusted life years (QALYs), costs of intervention and treatment, disease event costs, incremental cost-effectiveness ratio (ICER), and cost for the number needed to screen. Multiple scenario and sensitivity analyses were performed.

**Results:**

Compared with usual care, AI-integrated ECG was cost effective, with an incremental cost-effectiveness ratio of $27,858/QALY. The program remained cost effective even with a change in patient age and follow-up time duration, although the specific ICER values varied for these parameters. The program was more cost effective in outpatient settings (ICER $1651/QALY) than in inpatient or emergency room settings.

**Conclusion:**

Implementing AI-guided targeted screening for low EF in routine clinical practice was cost effective.

Approximately 1.4-2.2% of the general population develops asymptomatic left ventricular systolic dysfunction, defined as a left ventricular ejection fraction (EF) less than 40% without associated heart failure (HF) symptoms.^1^^,^^2^ Asymptomatic low EF is associated with an increased risk of developing symptomatic heart failure, leading to subsequent morbidity and mortality.^2^ Early detection of asymptomatic low EF can therefore support early treatment of this condition, which in turn has been shown to improve EF, delay the development of symptomatic heart failure, and ultimately reduce mortality.^3^

An artificial intelligence (AI) integrated electrocardiogram (ECG) (AI-ECG) was recently developed and validated to be an effective and convenient clinical decision aid to detect low EF and a cost effective screening method.^4^, ^5^, ^6^ In clinical practice, ECGs are performed for a variety of reasons. For this group of patients, the incorporation of AI algorithms into regular ECGs could help clinicians identify low EF and initiate appropriate early medical intervention. The ECG ai-guided screening for low ejection fraction (EAGLE) clinical trial (NCT04000087) found that AI-ECG enabled the early detection of low EF among 22,642 adult patients from 45 primary care clinics.^7^ However, whether large scale implementation of AI-ECG in routine practice is cost effective is unknown.

Therefore, the goal of this study was to explore the economic impact of AI-ECG by prospectively capturing the short-term outcomes (ie, low EF status) and simulating long-term outcomes (eg, disease development, mortality) and converting them into the patient burden and economic value. We, therefore, explored the cost-effectiveness of AI-ECG under various scenarios that might occur in routine clinical practice.

## Patients and Methods

### Study Design and Model Structure

**Figure 1:**
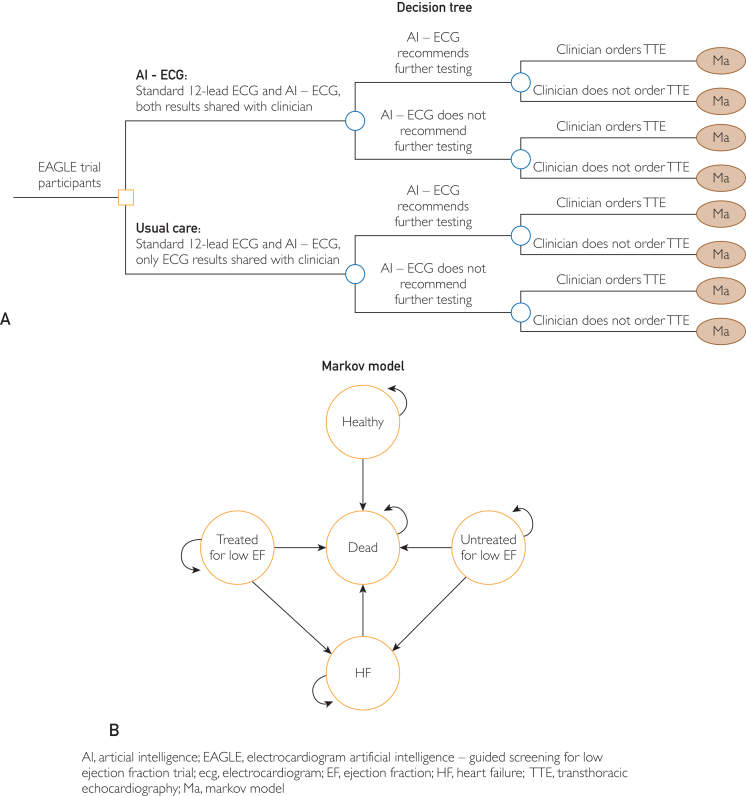
Model Structure. (A) Decision tree. (B) Markov model. AI-ECG, artificial intelligence integrated electrocardiogram; EAGLE, ECG AI-guided screening for low ejection fraction; ECG, electrocardiogram; EF, ejection fraction; HF, heart failure; Ma, Markov model; TTE, transthoracic echocardiogram.

We developed a decision analytic model with a short-term decision tree using results from the EAGLE trial and a lifetime Markov model to evaluate the cost-effectiveness of AI-ECG vs no AI-ECG. The decision tree structure is illustrated in Figure 1A, and the Markov model is illustrated in Figure 1B.

Our decision tree structure mirrors what occurred in the EAGLE trial. In the EAGLE trial, inpatient/outpatient patients with no signs or clinical diagnosis of HF underwent a standard 12-lead ECG between August 5, 2019, and March 31, 2020. As reported previously, an AI algorithm was embedded in the ECG interpretation, providing the probability of low EF to ordering clinicians who were part of a care team randomized to the intervention arm.^7^ On the basis of these results, recommendations on ordering a transthoracic echocardiogram (TTE) were provided, but the decision to pursue further work-up of potential low EF was left to the discretion and clinical judgment of the ordering clinician. The AI algorithm was applied to all patients, but results from the algorithm were only reported to the physicians in the AI-ECG-informed group; the results were not reported to the control group (no AI-ECG group).

Patients were then categorized as either AI-ECG positive (ie, recommending further testing for low EF) or AI-ECG negative (ie, no further testing needed). After AI-ECG results, patients were grouped on the basis of whether TTE was performed. We used the data collected from the EAGLE trial to determine the probability that a TTE was performed when AI-ECG results were either shared or not shared with the ordering clinician.

Our Markov model is in which we projected the long-term outcomes associated with early detection of low EF. Low EF in this economic evaluation was defined as EF ≤40%. The Markov model also included the long-term outcomes of those who did not have low EF and those who had low EF but were not diagnosed at an early stage.

It was difficult to determine whether a patient did indeed have low EF when TTE was not performed, as TTE is needed for diagnosing low EF. For patients who did not have TTE performed, we used the positive predictive value (PPV) of the AI-ECG to determine the likelihood that a patient had low EF. For patients whose AI-EGC recommended no further testing, we used the negative predictive value to estimate the rate of normal EF. The PPV and negative predictive value were identified from studies validating AI algorithms by Attia et al.^4^^,^^5^

For the patients who underwent TTE, the results of the TTE determined where patients entered the Markov model. Patients with TTE indicating low EF were assigned to the “low EF, treated” health state, where they remained until they developed symptomatic low EF or died. Patients with TTE indicating normal EF were assigned to the “normal EF” health state, where they remained until they died. Patients with no TTE but had low EF were assigned to the “low EF, untreated” health state, where they remained until they became symptomatic or died. Patients who became symptomatic would be denoted as moving to an HF state.

### Study Parameters

#### Decision Tree Probabilities

The probabilities utilized in the decision tree were obtained from the EAGLE trial (eg, the proportion of patients that underwent TTE and the proportion of patients identified to have low EF).

#### Markov Model Probabilities

The range and distribution of parameters for the Markov model were obtained from published randomized controlled clinical trials and meta-analyses (Table 1).^4^, ^5^, ^6^, ^7^, ^8^, ^9^, ^10^, ^11^, ^12^, ^13^, ^14^, ^15^ In the EAGLE trial, among 11,068 patients in the control group/usual care, 18.2% received TTE, and 0.6% were found to have EF≤40%; among the 11,573 patients in the intervention group, 19.2% received TTE, and 102 (0.9%) were found to have EF≤40%. Among either the intervention or control group, 6% were flagged by the AI as high-risk. Among these high-risk patients, 38.1% of the 664 control patients received an echo and of those who had TTE, 22.7% were diagnosed with EF≤40%; 49.6% of the 692 intervention patients received TTE and of those who had TTE, 22.1% were diagnosed with EF≤40%.^7^ These parameters from the prior EAGLE trial that tested the AI-ECG technology in routine practice were used in the model input as outlined in Table 1. Mortality was informed by the United States Center for Disease Control’s National Vital Statistics System.^16^ When low EF is identified and treated, clinical trials show that treatment decreases progression to symptomatic HF, rates of hospitalizations due to HF, and mortality associated with HF.^17^^,^^18^ Thus, patients who were diagnosed with low EF were treated with carvedilol and lisinopril and gained the benefits of early treatment. Patients with unidentified low EF did not receive the benefits of early treatment.

**Table 1:** Summary of Key Model Variable Inputs

Variable	Root definition	Range, or SE	Distribution	Reference
Cohort inputs				
Age (y) at ECG screening	63	(18, 102)	Table	^7^
Gender	54% Female	(0, 1)	Table	^7^
Time frame, y	70	-	Constant	Assumption
Costs		Range, or SE		
Cost of AI-ECG	100	(100, 300)	Constant	Assumption
Cost of regular ECG	50	50	Constant	^8^
Cost of Echo (TTE)	1801	1801	Constant	^9^
Cost of stress myocardial perfusion scan (SPECT)	2477	2477	Constant	^9^
Annual cost of unidentified low EF	0	0	Constant	Assumption
Event cost of HF	9001	93.5	Gamma	^6^
Annual cost of HF	5510	160.6	Gamma	^6^
Event cost of identified low EF	839	65.1	Gamma	^6^
Annual cost of identified low EF (medications: carvedilol and lisinopril)	2188	2188	Gamma	^6^
Annual cost at a healthy status	Age-based	(3591, 34,300)	Table	^10^
Transition probabilities		Range, SE, or alpha, beta		
Probability of developing HF from unidentified low EF-untreated	0.098	0.026	Beta	^11^
Probability of developing HF from unidentified low EF-treated	0.065	0.011	Beta	^11^
Additional risk of death with unidentified low EF from all causes untreated	3.3	1.1	Beta	^6^
Additional risk of death with unidentified low EF from all causes treated	2.9	0.9	Beta	^12^
Additional risk of death with HF from all causes	4.9	1.9	Beta	^5^
Probability of death from non-CVD causes	Age-based	(0.001, 0.381)	Table	^13^
Positive predictive value of AI-ECG	0.338	(3567, 6977)	Beta	^4^^,^^5^
Negative predictive value of AI-ECG	0.987	(41,762, 564)	Beta	^4^^,^^5^
Quality of life		SE		
QALY of unidentified low EF	0.855	0.005	Gamma	^14^
Event QALY change of HF	-0.024	0.004	Gamma	^15^
QALY of HF	0.673	0.046	Gamma	^14^
Event QALY change of identified low EF	-0.024	0.004	Gamma	^15^
QALY of healthy status	1	0.125	Gamma	Assumption
Trial outcome		Alpha, beta		
*Base-case AI-ECG*				
Proportion of AI-positive	0.060	(692, 10,881)	Beta	^7^
Proportion of Echocardiogram in AI-positive	0.496	(343, 349)	Beta	^7^
Proportion of Echocardiogram in AI negative	0.173	(1879, 9002)	Beta	^7^
Proportion of low EF by Echo in AI-positive	0.227	(78, 265)	Beta	^7^
Proportion of low EF by Echo in AI negative	0.013	(24, 1855)	Beta	^7^
Base-case no AI-ECG				
Proportion of AI-positive	0.060	(664, 10,404)	Beta	^7^
Proportion of Echocardiogram in AI-positive	0.381	(253, 411)	Beta	^7^
Proportion of Echocardiogram in AI negative	0.170	(1764, 8640)	Beta	^7^
Proportion of low EF by Echo in AI-positive	0.221	(70, 183)	Beta	^7^
Proportion of low EF by Echo in AI negative	0.008	(14, 1750)	Beta	^7^
Outpatient AI-ECG		(No sensitivity analysis was conducted)		
Proportion of AI-positive	0.040	-	Constant	^7^
Proportion of Echocardiogram in AI-positive	0.492	-	Constant	^7^
Proportion of Echocardiogram in AI negative	0.172	-	Constant	^7^
Proportion of low EF by Echo in AI-positive	0.200	-	Constant	^7^
Proportion of low EF by Echo in AI negative	0.013	-	Constant	^7^
Outpatient no AI-ECG		(No sensitivity analysis was conducted)		
Proportion of AI-positive	0.040	-	Constant	^7^
Proportion of Echocardiogram in AI-positive	0.305	-	Constant	^7^
Proportion of Echocardiogram in AI negative	0.167	-	Constant	^7^
Proportion of low EF by Echo in AI-positive	0.222	-	Constant	^7^
Proportion of low EF by Echo in AI negative	0.006	-	Constant	^7^

In probability sensitivity analysis, the standard deviation was used in defining distributions. A quarter of the range (1/4) was used as an estimation of standard deviation.

AI, artificial intelligence; AI-ECG, artificial intelligence integrated electrocardiogram; CVD, cardiovascular disease; ECG, electrocardiogram; EF, ejection fraction; HF, heart failure; SPECT, post-confirmatory test; TTE, transthoracic echocardiogram.

#### Assumptions

Assumptions were made on parameters when data were not available in the literature (Supplemental Table, available online at https://www.mcpdigitalhealth.org/). In the model, the patient groups were assumed to be at the same health level as the general population. Once low EF status was established, patients underwent standard therapy according to practice guidelines. The patient who was not identified to have low EF or other incidental findings was assumed to receive no treatment until the disease was identified in subsequent time cycles (in years). However, in the real world, a patient could get relevant treatments for other cardiovascular conditions than HF. Transthoracic echocardiogram is the gold standard for identifying low EF (100% sensitivity). There were no incidental findings from the TTE, excluding valvular heart disease and hypertrophic cardiomyopathy, which was due to 1) a small sample number or 2) not significantly different between the AI-ECG and the regular ECG group. The ECG was done in both arms for reasons other than HF; therefore, both arms had the cost of a standard ECG.

#### Costs

The cost of screening in the no AI-ECG group was assumed to be the cost of a standard 12-lead ECG ($50).^8^ The cost of screening for the AI-ECG group was assumed to be $100, accounting for an additional $50 cost to apply the AI-ECG algorithm on top of standard ECG ($50). This is an exploratory analysis, and a sensitivity analysis of a full range of costs was examined. The cost of TTE was assumed to be $1801.^13^ If the patient was identified to have low EF, there was an additional cost of $2477 for additional evaluation, such as the stress myocardial perfusion scan as a confirmatory test.^9^ For symptomatic HF, both event costs (ie, short-term hospital care) and annual long-term disease management costs (ie, cost of medications) were included and listed in Table 1. The cost was examined from the payer’s perspective (ie, Medicare).

#### Quality-Adjusted Life Years

The effectiveness of AI-ECG and no AI-ECG was calculated using quality-adjusted life years (QALYs), which is a composite value of combined quality of life (QoL) and life years. For each year of a healthy life, a patient was assigned 1 QALY. Each disease state (eg, low EF, treated) and disease event had a reduced QALY value, as detailed in Table 1.

#### Cost-Effectiveness Analysis

For our cost-effectiveness analysis, we followed the guidelines outlined by the 2nd Panel on Cost-Effectiveness in Health and Medicine.^19^ Incremental cost-effectiveness ratios (ICERs) were calculated and compared with a willingness-to-pay (WTP) of $100,000. Strategies with better clinical outcomes and ICERs within the threshold of WTP were defined as the preferred strategy over the comparator. Lifetime costs, incremental costs, QALYs gained, and incremental QALYs were also calculated. Scenarios analyses were conducted according to the patient’s age and follow-up time frame.

#### Analysis of Uncertainty

Cost-effectiveness under different scenarios was examined, including age, length of follow-up, whether the AI-ECG yielded a positive (high-risk of low EF) or negative (low risk of low EF) result, and outpatient setting. The oldest age in the scenario analysis was 80 years since the life expectancy in the United States was 78 years old. The EAGLE trial reported that the differences were the greatest among patients whose AI-ECG results were positive and those in the outpatient setting.^7^ A hypothetic scenario that all the patients who were AI-ECG positive were simulated in this model, which allowed us to explore the potential maximum benefit AI-ECG could bring if the providers were notified of the positive results among an extremely high-risk patient population (ie, all the patients were AI-positive, with a small proportion of false negative results corresponding to the PPV of AI-ECG). These parameters with the greatest clinical significance were examined using one-way sensitivity analyses. We further simulated 1000 theoretical trials to investigate the uncertainty of this result using probabilistic sensitivity analysis. The distribution of ICERs and cost-effectiveness acceptability curve were reported.

In the primary analysis, we assumed that once diagnosed, all patients would receive treatment. In the prior pragmatic trial, we found 95.3% of patients received at least 1 prescription, with 79.1% receiving angiotensin-converting enzyme inhibitors or angiotensin II receptor blockers, and 91.3% receiving beta-blockers. However, guideline-directed medical therapy can be suboptimal. In a sensitivity analysis, we assumed 50% of these diagnosed patients were treated.

#### General Model Information

The analysis used a 3% annual discount rate for both costs and QALYs. Costs were converted into 2020 US dollars using the US Bureau of Labor Statistics medical care in US city average.^20^ The Markov model was set to annual cycles. The model was constructed and analyzed using TreeAge Pro Healthcare 2020 software (TreeAge Software, Inc).

## Results

### Base-Case Analysis

The study population included 22,641 patients aged 18 years and older with a median age of 63 (range, 18-102) years (age distribution detailed in Supplemental Figure 1, available online at https://www.mcpdigitalhealth.org/) with no prior symptoms or findings suggesting HF. The patient population was composed of 53.9% women. The results of the base-case analysis are presented in Table 2. The average lifetime health care costs for non-AI-ECG patients were $224,564, whereas the lifetime costs for patients in the AI-ECG group were $224,950; thus, on average, AI-ECG was associated with increased health care costs of $386. The no AI-ECG group gained 14.83 QALYs, whereas the AI-ECG group gained 14.85 QALYs, with a net gain of 0.014 QALYs as a result of AI-ECG use. At a WTP of $100,000/QALY, AI-ECG was cost effective compared with no AI-ECG with an ICER of $27,858/QALY.

**Table 2:** Cost-Effectiveness of AI-ECG vs no AI-ECG Among Patients With no Previous Diagnosis of Heart Failure (2020 USD)^a^

Cost-effectiveness by QALY	Costs, $	QALYs, n	Incremental costs, $	Incremental QALYs, n	ICER, $/QALY	Conclusion^b^	LYs, n
Base-case cohort							
No AI-ECG	224,564	14.83	-	-	-	-	19.72
AI-ECG	224,950	14.85	386	0.0139	27,858	Cost effective	19.73
Scenario analysis							
Age (y)							
20 y old							
No AI-ECG	167,865	26.16	-	-	-	-	52.54
AI-ECG	168,039	26.17	174	0.0134	13,007	Cost effective	52.56
40 y old							
No AI-ECG	217,656	21.26	-	-	-	-	35.11
AI-ECG	218,001	21.27	345	0.0169	20,419	Cost effective	35.13
60 y old							
No AI-ECG	224,190	14.97	-	-	-	-	20.00
AI-ECG	224,570	14.99	380	0.0137	27,713	Cost effective	20.02
70 y old							
No AI-ECG	193,781	11.24	-	-	-	-	13.31
AI-ECG	194,132	11.25	350	0.0102	34,196	Cost effective	13.32
80 y old							
No AI-ECG	146,749	7.20	-	-	-	-	7.40
AI-ECG	147,081	7.21	331	0.0092	36,213	Cost effective	7.41
Time frame (y)							
10 y							
No AI-ECG	96,835	8.05	-	-	-	-	8.19
AI-ECG	97,033	8.06	198	0.01	35,698	Cost effective	8.19
20 y							
No AI-ECG	174,808	12.68	-	-	-	-	15.22
AI-ECG	175,102	12.69	295	0.01	30,095	Cost effective	15.23
50 y							
No AI-ECG	224,557	14.83	-	-	-	-	19.71
AI-ECG	224,943	14.85	386	0.01	27,858	Cost effective	19.73
AI-positive only patients							
No AI-ECG	173,208	12.64	-	-	-	-	17.10
AI-ECG	224,564	14.83	51,356	2.19	23,435	Cost effective	19.72
Patients with underlying low EF							
No AI-ECG	25,590	6.63	-	-	-	-	10.00
AI-ECG	26,204	6.67	614	0.05	13,136	Cost effective	10.08
Outpatient							
No AI-ECG	190,412	14.12	-	-	-	-	20.52
AI-ECG	190,422	14.13	10	0.01	1651	Cost effective	20.54

Costs and QALYs were per person over the time frame of 70 years for base-case, low EF was defined as EF≤40%.

a AI-ECG, artificial intelligence integrated electrocardiogram; ECG, electrocardiogram; EF, ejection fraction; LY, life-year; QALY, quality-adjusted life-year.

b Conclusion was made based on willingness-to-pay = $100,000.

### Scenario Analysis

We examined scenarios covering a range of ages and follow-up times. As patient age changed from 20 years to 80 years old, the costs of both no AI-ECG and AI-ECG varied: the highest cost increase was among patients aged 60 years ($224,190 no AI-ECG vs $224,570 AI-ECG), and the QALY decreased with age (from 26.16 QALYs to 7.20 QALYs for no AI-ECG vs 26.17 QALYs to 7.21 QALYs for AI-ECG). Artificial intelligence integrated electrocardiogram was more cost effective for younger age groups. Incremental cost-effectiveness ratio increased from $13,007/QALY at age 20 years to $36,213/QALY at age 80 years. The costs and QALY increased with the study follow-up time frame. Results suggested that the cost-effectiveness was lowest when the follow-up duration was 10 years (ICER=$35,698/QALY) and greatest when the follow-up was 50 years (ICER=$27,858/QALY).

We examined scenarios covering a range of prediction and treatment possibilities. The EAGLE trial suggested that among the AI-indicated positive cases, physicians were more likely to order TTE when provided AI-positive results.^7^ Under this scenario of AI reported positive only (regardless of whether TTE was performed or not), AI-ECG was more effective compared with no AI-ECG (ICER= $23,435/QALY). Compared with no AI-ECG, the QALYs gained from AI-ECG were the highest compared with other scenarios or the full cohort (2.19 QALYs). Among the patients with low EF, AI-ECG was cost effective (ICER=$13,136/QALY) by improving 0.05 QALYs and $614 incremental costs. When conducted in an outpatient setting, AI-ECG was preferred (smaller ICER) over no AI-ECG by increasing cost by $10 ($190,412 vs $190,422) although slightly improving QoL by 0.01 QALYs (14.13 QALYs vs 14.12 QALYs), which led to an ICER of $1651/QALY.

### One-Way Sensitivity Analyses

**Figure 2:**
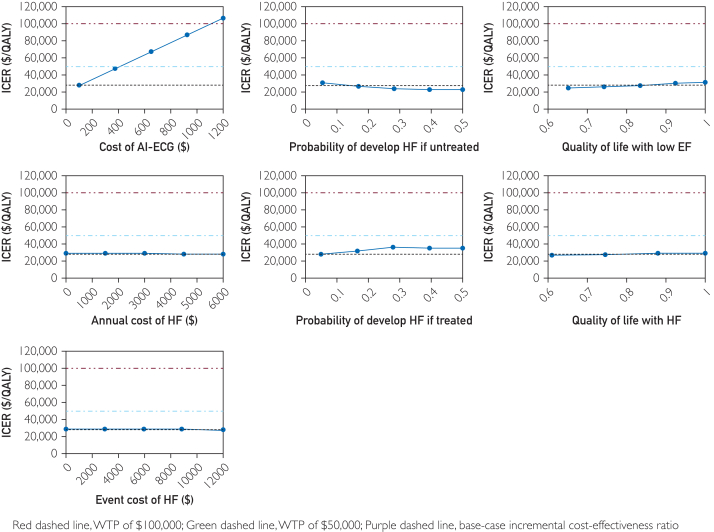
Results from one-way sensitivity analysis. AI-ECG, artificial intelligence integrated electrocardiogram; EF, ejection fraction; HF, heart failure; ICER, incremental cost-effectiveness ratios; QALY, quality-adjusted life years; WTP, willingness-to-pay.

One-way sensitivity analyses were performed on the parameters associated with costs, utility, and transition probabilities. Ten variables with the most clinical relevance in patient management are reported in Figure 2. Overall, as expected, increases in the cost of testing were related to increased ICER (less cost effective). The increase in the annual cost of HF and event cost of HF were related to decreased ICER (more cost effective). The increase in probabilities of developing HF if treated or untreated were all related to ICER but in non-linear patterns. The increase in QoL with asymptomatic left ventricular dysfunction and HF was related to a slight increase in ICERs. More importantly, the one-way sensitivity analysis reported that even with - substantial increases in the costs of AI-ECG, TTE, post-confirmatory testing, and the annual costs of managing low EF, AI-ECG remained cost effective with ICERs that did not cross the $50,000 or $100,000 WTP threshold until the aforementioned costs were much higher than what would be expected in the real world.

### Probabilistic Sensitivity Analyses

**Figure 3:**
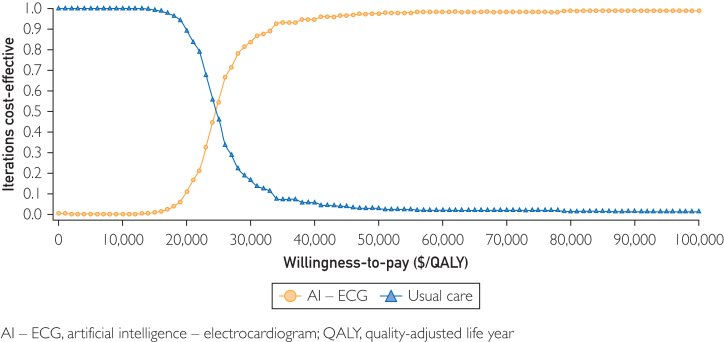
Results from probabilistic sensitivity analysis. AI-ECG, artificial intelligence integrated electrocardiogram; QALYs, quality-adjusted life years.

We performed 1000 samples of 10,000 patients where the input parameters were varied simultaneously, along with the prespecified distributions for the underlying parameters. The cost effectiveness acceptability curve was examined (Figure 3). When the WTP was below $27,500/QALY, no AI-ECG was cost effective in over 50% of the simulations. When the WTP was above $27,500/QALY, AI-ECG was cost effective in over 50% of simulations. The cost-effectiveness plane is illustrated in Supplemental Figure 2 (available online at https://www.mcpdigitalhealth.org/). At the WTP of $100,000, AI-ECG was cost effective 79.5% of the time. In 0.9% of the simulated trials, AI-ECG improved QoL but was not cost effective (ICER>$100,000/QALY).

In the sensitivity analysis where we assumed half of the diagnosed patients would be treated, implementing AI-ECG would still be cost effective, with an ICER of $23,328/QALY.

## Discussion

The current study provided insights into the adoption of AI algorithms as clinical decision-support tools. The study results suggested that AI-ECG could be implemented system-wide, regardless of age, but priority should be given to younger patients and those in outpatient settings. The study highlighted that AI-ECG would be cost effective regardless of the common threshold level used. The most widely accepted WTP threshold for cost-effectiveness studies in the United States is $100,000/QALY, with some studies using a lower threshold of $50,000/QALY.^21^^,^^22^ The payer’s WTP threshold was below the most accepted WTP, making AI-ECG the preferred strategy in most cases.

This cost-effectiveness study extended the findings from a series of prior studies on this algorithm,^4^, ^5^, ^6^, ^7^^,^^23^^,^^24^ offering a patient-centered economic evaluation over a longer period. The current evaluation found that the AI-ECG would cost around $30,000 per QALY gained, compared with approximately $50,000 in a prior study.^6^ Both studies concluded that using AI-ECG for early detection of low EF is cost effective, despite differences in exact dollar amounts due to various factors.

The current study differed from the prior one by leveraging parameters from a pragmatic trial that implemented the technology on a large scale in routine practice. Unlike the prior study, which assumed everyone with a positive AI-ECG result would receive TTE, the pragmatic trial reported that only half of the patients did so, reflecting real world practice. Additionally, the current study did not consider the cost of ECGs, as it assumed AI would be applied to existing ECGs ordered for other clinical indications. The definition of low EF also differed, with the current study considering EF≤40% compared with EF≤35% in the prior study.

The current analysis excludes the technology’s nominal upfront cost. The incremental cost for Mayo Clinic, with its robust data infrastructure, was negligible. Although licensing costs for this AI algorithm may be incurred, they are expected to be low, but other health systems could incur costs for additional computational resources, storage, and staff training. These expenses are challenging to quantify given their potential system-wide benefits, such as enhanced security and operational efficiency. Nevertheless, on the basis of our study’s findings, we derived a cost-effectiveness ratio of $27,858/QALY with a 0.0139 QALY increase. The lifetime difference in cost between AI-ECG and no AI-ECG was an additional $386 per person in the AI-ECG intervention. Should the acceptable cost-effectiveness threshold be $50,000/QALY, the lifetime difference in cost between AI-ECG and no AI-ECG was an additional $695 per person, leaving a margin of $309 to absorb technology costs. At a $100,000/QALY threshold, this margin increases to $1004 per patient.

Furthermore, dedicated resources will be needed for monitoring the algorithm’s performance, identifying reasons for performance shifts if there are any, and potentially fine-tuning the algorithm. Different kinds of algorithms, depending on how they were trained and what data the algorithms are run on, have different propensities for performance shifts. However, regardless of the propensity, dedicated resources to support such monitoring and retraining, potentially as part of the licensing cost, will need to be considered during the initial implementation phase and re-evaluated regularly during long-term implementation.

The study has a few limitations. First, although this study used clinical trial results, the lifetime Markov was based on model prediction with assumptions, which might or might not hold. These assumptions were made according to the most likely scenarios using the judgments from experienced clinicians.

Second, the use of single-trial results could lead to less external validity, especially with regard to patient demographic characteristics and practice patterns. The study population in the trial involved an integrated health care system in the upper Midwest in the United States with less racial and ethnic diversity and fewer urban areas compared with the general US population. However, this clinical trial was designed pragmatically in diverse clinical settings, including tertiary medical centers as well as rural clinics.

Third, in this economic evaluation, low EF was defined as EF≤40% because the guideline recommendations are stronger and the impact on subsequent clinical outcomes is clearer. However, as reported in the EAGLE trial, the AI-ECG could also enable early diagnosis of EF between 40% and 50%, also known as HF with mid-ranged EF or HF with mildly reduced EF. With emerging therapies for patients with HF with mildly reduced EF, the actual cost-effectiveness could be greater than what was estimated in the present analysis.

## Conclusion

Compared with no AI-ECG, the use of AI-ECG to detect low EF was cost effective, with the potential to improve QoL, particularly in younger patients and patients seen in an outpatient setting. Further investigation in different clinical and demographic settings should be performed to evaluate the potential use of this tool in broader clinical screening for low EF.

## Potential Competing Interests

Dr Borah has consulted Exact Sciences and Boehringer Ingelheim in the last 3 years on subject matter unrelated to the current submission. Dr McCoy has received support from NIDDK, PCORI, and AARP and also serves as a consultant to Emmi (Wolters Kluwer) on the development of patient education materials related to prediabetes and diabetes. Along with Mayo Clinic, Drs Attia, Friedman, and Noseworthy have filed patents related to the application of artificial intelligence to the electrocardiogram for diagnosis and risk stratification and have licensed several artificial intelligence integrated electrocardiogram algorithms to Anumana. This includes an artificial intelligence integrated electrocardiogram algorithm for the detection of heart failure studied in the current manuscript. Dr. Lopez-Jimenez serves as a member of the advisory board for Anumana, an AI company that has licensed this algorithm, he is a coinventor of this algorithm and may benefit financially from the commercialization of this technology. In the previous 3 years of this submitted work, Dr. Pellikka had research support from Ultromics and the American Society of Echocardiography Foundation, with payment to Mayo Clinic. Dr. Rushlow serves as Mayo Clinic Principal Investigator for the GUARD AF study sponsored by Bristol Meyers Squibb and received no direct compensation. None of the other authors have any potential conflicts of interest with respect to the authorship and/or publication of this article. None reported any financial relationships with any organizations that might have an interest in the submitted work in the previous 3 years. Given their roles as Editor and Editorial Board Member, Drs Francisco Lopez-Jimenez, Paul A. Friedman, and Zachi I. Attia were not involved in the peer review of this article and have no access to information regarding its peer review. Full responsibility for the editorial process for this article was delegated to an unaffiliated Editor.

## Ethics Statement

Mayo Clinic Institutional Review Board determined that this study was exempt from review as it used preexisting, de-identified data.
