# Supplementary material for: Cost-Effectiveness of Artificial Intelligence-Enabled Electrocardiograms for Early Detection of Low Ejection Fraction: A Secondary Analysis of the Electrocardiogram Artificial Intelligence-Guided Screening for Low Ejection Fraction Trial
Source: Mayo Clin Proc Digit Health. 2024 Oct 26;2(4):620–31. doi: 10.1016/j.mcpdig.2024.10.001 (PMC11975989; doi:10.1016/j.mcpdig.2024.10.001)
Supplement: Supplemental Table and Figures [file mmc1.pdf]

Supplemental Table. Model Assumptions

	Population Selection
1	The patient groups were assumed to only have the disease comorbidities listed in the study (ALVSD or CHF). Otherwise, the patients were assumed to be at the same health level of general population.
2	Once low EF is diagnosed, patients underwent standard therapy which state that they start on carvedilol and lisinopril.
3	The patient who was not identified to have low EF or other incidental findings was assumed to receive no treatment until symptoms occur in the following time cycles (in years).
	**Disease States**
4	The major disease state following long-term low EF is chronic heart failure (CHF). [1]
5	Patient groups who underwent echocardiogram would all have low EF identified. Therefore, for these groups of patients, the unidentified low EF status was 0.
6	The identified low EF in the no echocardiogram groups was defined as any previous findings of low EF or LVSD from previous visits for all causes. This was estimated from a US population level. [1]
7	The incidental findings from the echocardiogram, including valvular heart disease and hypertrophic cardiomyopathy were not included in this model due to 1) small sample number, or 2) not significant between the AI-ECG and the regular ECG group.
8	The quality of life of CHF were obtained from patients with NYHA class III and above. Patients with heart failure that was NYHA class II and below were assumed to have very mild symptoms that didn't affect their quality of life.
9	Patients with ALVSD were assumed to have no symptoms or very mild symptoms so relevant treatments were given. However, in the real world, patient could get relevant treatments for other cardiovascular conditions than heart failure.
10	Patients who didn't develop ALVSD, LVSD or CHF were assumed to be healthy and have quality of life = 1.
	**Limited Information**
11	We assumed that all the CHF were HFrEF (heart failure with reduced ejection fraction) due to limited information on HFpEF (heart failure with preserved EF). The reality is that this direct transition is probably unlikely, unless you are older and then you develop HFpEF. This complexity was out of the scope of this study which is focusing on low EF.
12	The AI-informed echocardiogram -performed group had the rates of low EF closest to the true rates in this patient population.
	**Model Simplicity**
13	Echocardiogram is a simple and widely available way to diagnose low EF condition (Sensitivity =1).

Reference:

1. Wang TJ, Evans JC, Benjamin EJ, Levy D, LeRoy EC, Vasan RS. Natural history of asymptomatic left ventricular systolic dysfunction in the community. Circulation 2003;108:977-82.

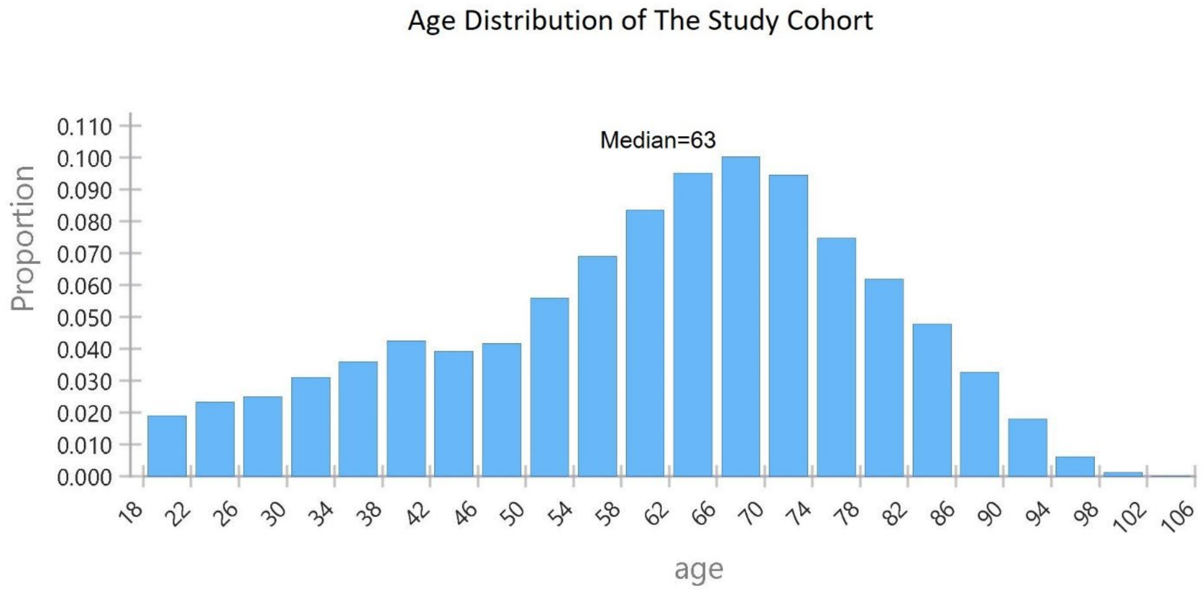

Supplemental Figure 1: Age distribution of the EAGLE population

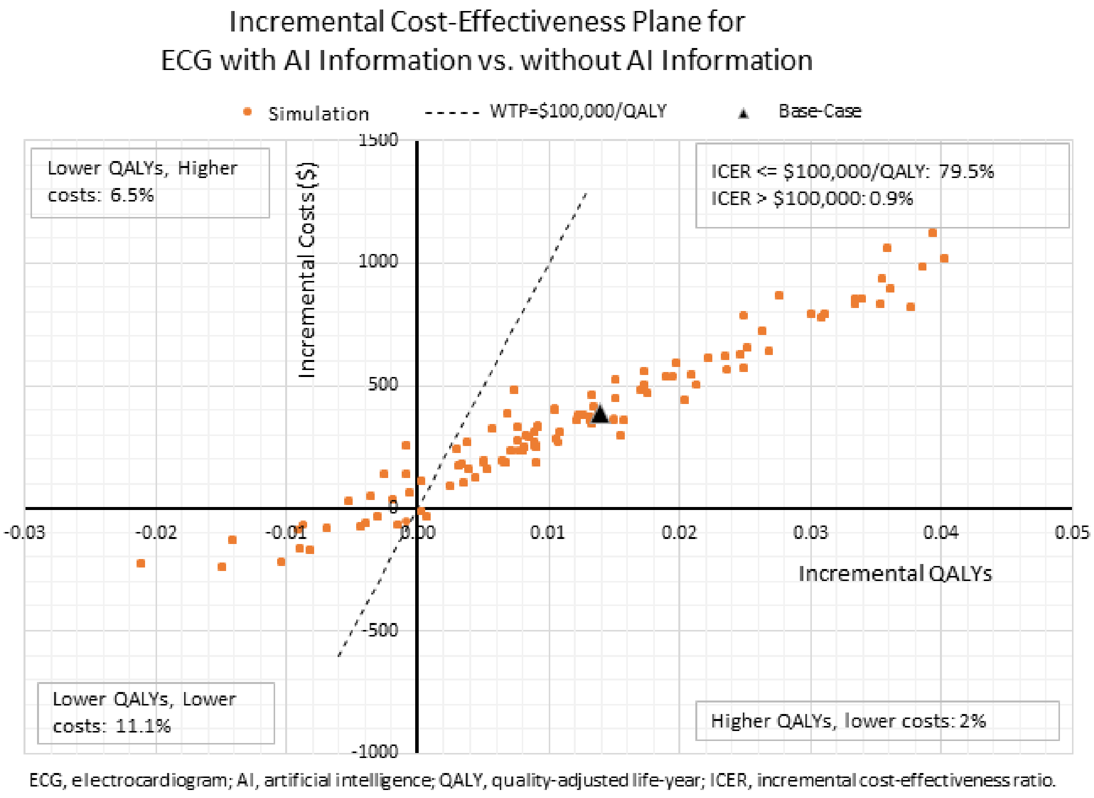

Supplemental Figure 2: Incremental Cost-Effectiveness Plane for AI-ECG versus no AI-ECG
